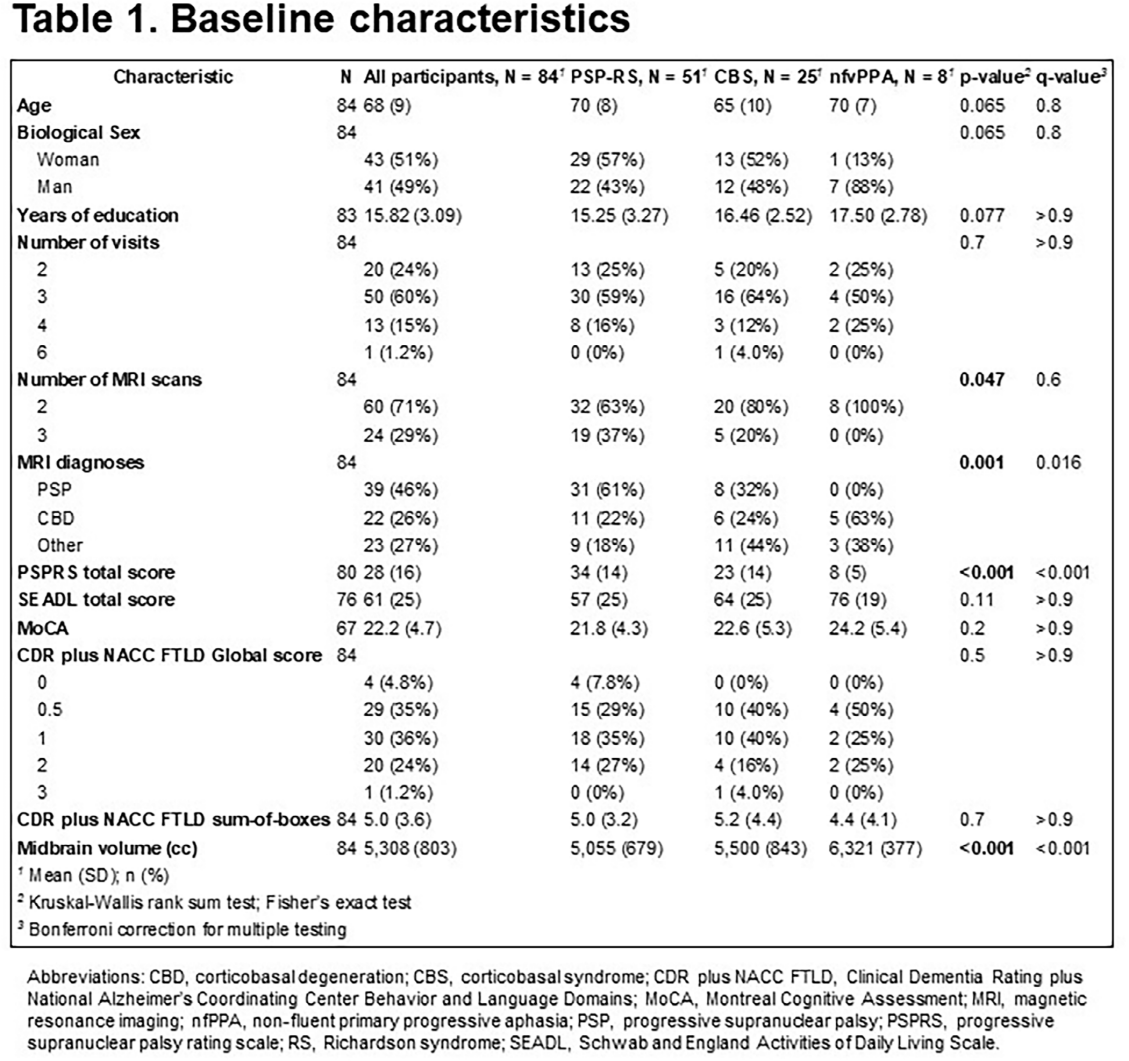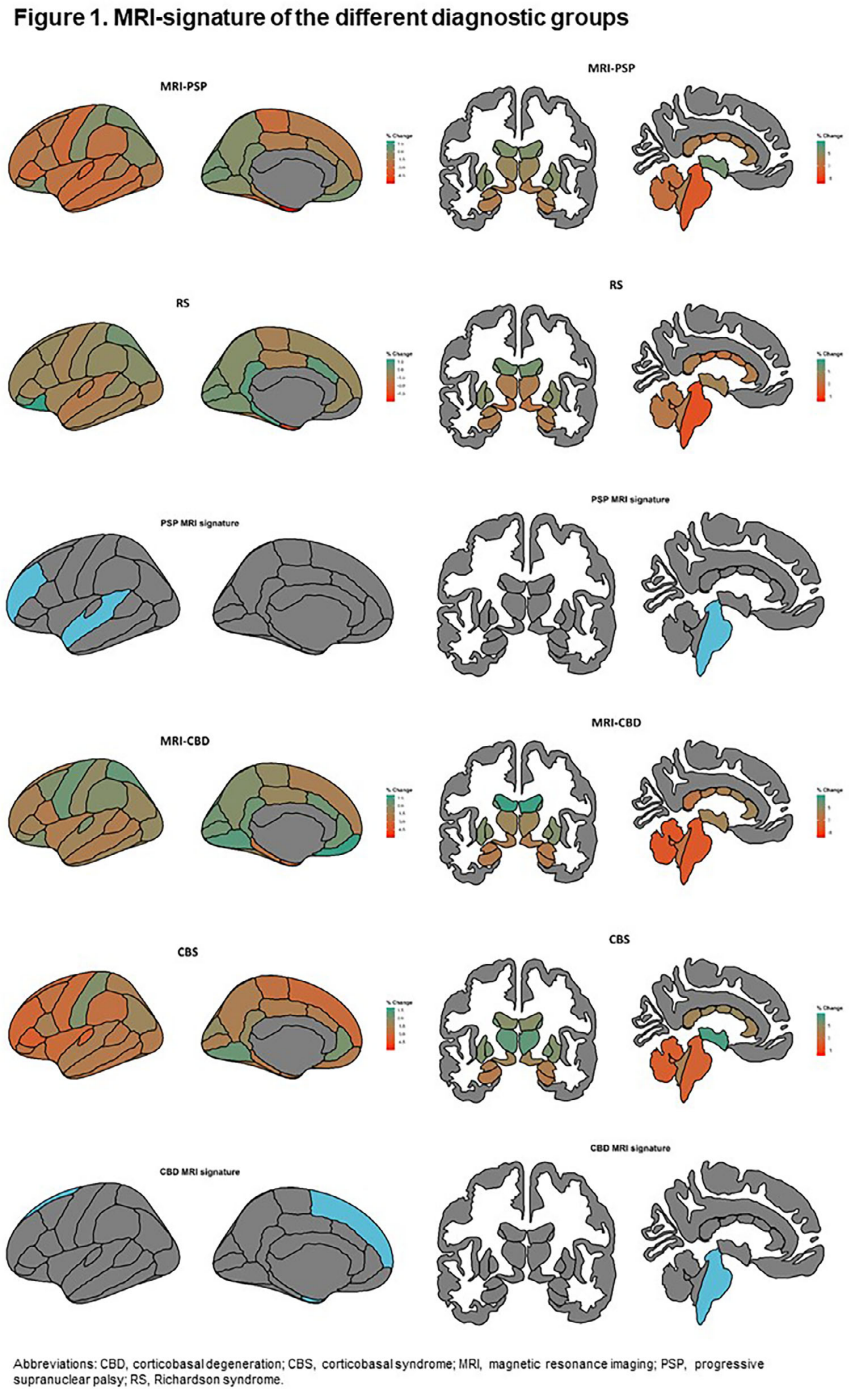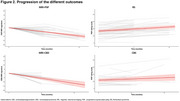# Exploring the role of MRI to optimize the design of clinical trials for progressive supranuclear palsy and corticobasal degeneration

**DOI:** 10.1002/alz70856_104263

**Published:** 2025-12-24

**Authors:** Jesús Garcia Castro, Lídia Vaqué‐Alcázar, Lawren VandeVrede, Michael C. Donohue, Hilary W. Heuer, Rema Raman, Sara Rubio‐Guerra, Judit Selma‐Gonzalez, Alejandra O. Morcillo‐Nieto, Oriol Dols‐Icardo, Alexandre Bejanin, Olivia Belbin, Juan Fortea, Daniel Alcolea, Maria Carmona‐Iragui, Isabel Barroeta, Miguel A Santos‐Santos, María Belén Sánchez‐Saudinós, Isabel Sala, Julio C. Rojas, Anne‐Marie Wills, Eden V. Barragan, Irene Litvan, Adam L. Boxer, Alberto Lleó, Ignacio Illán‐Gala

**Affiliations:** ^1^ Sant Pau Memory Unit, Hospital de la Santa Creu i Sant Pau ‐ Biomedical Research Institute Sant Pau ‐ Universitat Autònoma de Barcelona, Barcelona, Barcelona, Spain; ^2^ Sant Pau Memory Unit, Hospital de la Santa Creu i Sant Pau ‐ Biomedical Research Institute Sant Pau ‐ Universitat Autònoma de Barcelona, Barcelona, Spain; ^3^ Memory and Aging Center, UCSF Weill Institute for Neurosciences, University of California San Francisco, San Francisco, CA, USA; ^4^ Alzheimer's Therapeutic Research Institute, University of Southern California, San Diego, CA, USA, San Diego, CA, USA; ^5^ Memory and Aging Center, Weill Institute for Neurosciences, University of California, San Francisco, San Francisco, CA, USA; ^6^ Alzheimer's Therapeutic Research Institute, University of Southern California, San Diego, CA, USA; ^7^ Sant Pau Memory Unit, Hospital de la Santa Creu i Sant Pau, Institut de Recerca Sant Pau ‐ Universitat Autònoma de Barcelona, Barcelona, Spain; ^8^ Sant Pau Memory Unit, Hospital de la Santa Creu i Sant Pau, Biomedical Research Institute Sant Pau, Universitat Autònoma de Barcelona, Barcelona, Spain; ^9^ Sant Pau Memory Unit, Department of Neurology, Hospital de la Santa Creu i Sant Pau, Biomedical Research Institute Sant Pau ‐ Universitat Autònoma de Barcelona, Barcelona, Spain; ^10^ Sant Pau Memory Unit, Hospital de la Santa Creu i Sant Pau ‐ Biomedical Research Institute Sant Pau ‐ Universitat Autònoma de Barcelona, Barcelona, Cataluña, Spain; ^11^ Memory and Aging Center, Department of Neurology, Weill Institute for Neurosciences, University of California, San Francisco, San Francisco, CA, USA; ^12^ Massachusetts General Hospital, Boston, MA, USA; ^13^ University of California, San Francisco, San Francisco, CA, USA; ^14^ University of California, San Diego, La Jolla, CA, USA

## Abstract

**Background:**

Progressive supranuclear palsy (PSP) and corticobasal degeneration (CBD) are severe neurodegenerative disorders lacking disease‐modifying treatments and validated biomarkers. Clinical trials face challenges due to phenotypic overlap and imperfect clinicopathological correlations. MRI‐derived models have shown to accurately predict PSP and CBD pathology in a large autopsy‐confirmed cohort (Illán‐Gala et al., *JAMA Network Open*, 2022). This study examines how participant selection based on MRI models and imaging outcomes impacts sample size estimations in hypothetical clinical trials.

**Method:**

Eighty‐four participants from the 4 Repeat Tauopathy Neuroimaging Initiative (4RTNI) with baseline and longitudinal MRI data and clinical assessments were included. Diagnoses comprised Richardson syndrome (RS, 61%) and corticobasal syndrome (CBS, 30%) without Alzheimer's disease. MRI‐derived models predicted PSP, CBD, or other pathologies (MRI‐PSP, MRI‐CBD, MRI‐Other) according to baseline MRI. Cortical thickness and volume measures were derived from MRI data using Freesurfer and employed to identify an optimal MRI‐signature of regions showing the highest effect size on atrophy over 12 months using linear mixed‐effects models. Disease progression was also measured with PSP Rating Scale (PSPRS). Sample sizes required to detect a 30% reduction in mean change at 12 months were calculated for hypothetical clinical trials.

**Result:**

MRI predicted PSP, CBD, and other pathologies in 46%, 26%, and 27% of participants, respectively. Among RS diagnoses, 31 (61%) were classified as MRI‐PSP; among CBS, 6 (24%) were MRI‐CBD. MRI‐signature regions for PSP progression included midbrain, superior‐temporal, and rostral‐middle‐frontal thickness. For CBD, key regions included midbrain and pons volumes, superior‐frontal, and entorhinal thickness.

In a hypothetical PSP trial, selection criteria based on clinical diagnosis required 336 participants using PSPRS as outcome, while MRI‐based diagnosis with MRI‐signature as outcome reduced the sample size to 121 (64% decrease). For a CBS trial, the sample size reduced from 1301 participants if inclusion was based on clinical diagnosis to 160 using MRI‐based selection and outcomes.

**Conclusion:**

Selecting participants with increased diagnostic certainty for PSP and CBD based on baseline MRI, combined with using MRI measures as outcomes could enhance the efficiency of future phase 2 clinical trials for 4R tauopathies. We plan to replicate these results in the Davunetide trial cohort.